# Testing the Dose Addition Hypothesis: The Impact of Pyrethroid Insecticide Mixtures on Neurons

**DOI:** 10.1289/ehp.119-a399a

**Published:** 2011-09-01

**Authors:** Kris S. Freeman

**Affiliations:** Kris S. Freeman has written for *Encarta* encyclopedia, NIH, ABCNews.com, and the National Park Service. Her research on the credibility of online health information appeared in the June 2009 *IEEE Transactions on Professional Communication*.

Pyrethroid insecticides are used extensively in agriculture and in homes to control fleas, cockroaches, bedbugs, and other insects. In a new *in vitro* study researchers tested the hypothesis that mixtures of pyrethroids have a dose-additive effect—that is, that pyrethroids as a chemical group produce toxicity in mammals via a common mode of action and that the combined toxicity of a pyrethroid mixture reflects the sum of its constituents’ toxicities [*EHP* 119(9):1239–1246; Cao et al.]. Using increased sodium ion influx as a specific functional measure of toxicity, the researchers found that effects of a mixture of commonly used pyrethroids were consistent with a dose-additive effect on mammalian neurons.

Pyrethroids act on the nervous system by disrupting the normal function of voltage-gated sodium channels (VGSCs), which control the influx of sodium ions into neurons to transmit nerve signals. When VGSCs open, the influx of sodium generates the nerve signal; when they close, the electrical signal halts abruptly. Pyrethroids bind to VGSCs and delay their closing, which causes repetitive nerve stimulation that can lead to muscle tremors as well as interfere with the ability of the channels to respond to stimulation.

Previous research demonstrated that a mixture of 11 pyrethroids had a dose-additive effect on the rat nervous system, decreasing the animals’ motor activity at doses below the threshold dose of each constituent compound. The authors of the current study exposed neurons cultured from the cerebral cortices of embryonic mice to the same 11 pyrethroids, then examined how VGSCs responded. To measure sodium influx without disrupting cell function, neurons were treated with a sodium-sensitive dye that fluoresced when VGSCs were open.

Seven of the pyrethroids tested increased sodium influx in a dose-dependent manner (from highest to lowest potency: deltamethrin, *S*-bioallethrin, β-cyfluthrin, λ-cyhalothrin, esfenvalerate, telfluthrin, fenpropathrin). Cypermethrin and bifenthrin had only a marginal effect on sodium influx, whereas permethrin and resmethrin had no effect. Despite these variations in activity, the results when neurons were exposed to all 11 compounds together were consistent with a cumulative, dose-additive effect on neuronal sodium influx.

People are commonly exposed to low doses of pyrethroid mixtures, which tend to persist as residues on treated surfaces and in household dust. Currently the U.S. Environmental Protection Agency is determining whether a cumulative dose-additive model is appropriate to evaluate potential human health effects of pyrethroid mixtures. This model shows that simultaneous exposure to multiple compounds will produce an effect that is consistent with additivity at the VGSC molecular target.

